# Whole-Lesion CT Texture Analysis as a Quantitative Biomarker for the Identification of Homogeneous Renal Tumors

**DOI:** 10.3390/life12122148

**Published:** 2022-12-19

**Authors:** Xiaoyan Meng, Shichao Li, Cui Feng, Daoyu Hu, Zhen Li, Yonghua Niu

**Affiliations:** 1 Department of Radiology, Tongji Hospital, Tongji Medical College, Huazhong University of Science and Technology, Wuhan 430074, China; 2 Department of Pediatric Surgery, Tongji Hospital, Tongji Medical College, Huazhong University of Science and Technology, Wuhan 430074, China

**Keywords:** renal tumor, computed tomography, texture analysis

## Abstract

Renal tumors are very common in the urinary system, and the preoperative differential diagnosis of homogeneous renal tumors remains a challenge. This study aimed to evaluate the feasibility of the whole-lesion CT texture analysis for the identification of homogeneous renal tumors including clear cell renal cell carcinoma (ccRCC), chromophobe RCC (chRCC), and renal oncocytoma (RO). This retrospective study was approved by our local IRB. Contrast-enhanced CT examination was performed in 128 patients and histopathologically confirmed ccRCC, chRCC, and RO. The one-way ANOVA test with Bonferroni corrections was used to compare the differences, and the receiver operating characteristic (ROC) curve analysis was applied to determine the diagnostic efficiency. The whole-lesion CT histogram analysis was used to demonstrate significant differences between ccRCC and chRCC in both arterial and venous phases, and the entropy demonstrated excellent performance in discriminating these two types of tumors (AUCs = 0.95, 0.91). The inhomogeneity of ccRCC was significantly higher than that of RO both in arterial and venous phases. The entropy of chRCC was significantly lower than that of RO, and the kurtosis and entropy yielded high sensitivity (91%) and moderate specificity (74%) in the arterial phase. The whole-lesion CT histogram analysis could be useful for the differential diagnosis of homogeneous ccRCC, chRCC, and RO.

## 1. Introduction

Renal tumors are very common in the urinary system. Approximately 90% of renal tumors are renal cell carcinoma (RCC), which originates from renal cortical cells, and the three main types are clear cell RCC (ccRCC, 75%), papillary RCC (pRCC, 15%), and chromophobe RCC (chRCC, 5%). The remaining solid renal tumors include renal oncocytoma (RO), angiomyolipoma, adenoma, etc. [1]. Accurate preoperative diagnosis for the renal tumor is very important for postoperative prognosis evaluation, surgical method selection, and follow-up observation.

Clear cell tumors are commonly hyper-vascular and may exhibit coagulative tumor necrosis [2]. On imaging, ccRCC often presents with hemorrhage and necrosis, which are related to tumor grade [3,4]. Without these changes, accurate diagnosis is still challenging [5,6,7]. In addition, chRCC shows lower enhancement than ccRCC and is more homogenous but has a large overlap with ccRCC in enhancement features [6]. RO is the second common benign renal tumor. It was reported that RO and chRCC have a strong overlap in imaging findings as they originate from the same cell origin, namely the intercalated cells of the collecting duct [8,9,10]. Furthermore, chRCC is a malignant tumor with a mortality rate of 10%, while RO is benign and can be misdiagnosed as chRCC because of its nuclear atypia [2,11]. Therefore, accurate preoperative diagnosis is crucial.

CT texture analysis (CTTA) has been introduced to assess tumor heterogeneity by analyzing the distribution and association of pixel or voxel gray levels in the image, enabling detection of tissue differences that are invisible to the naked eye. It has been widely used in tumor diagnosis, response evaluation, and histology prediction [8,12,13]. In the renal tumor, CTTA has been a promising method to distinguish ccRCC from pRCC and to predict Fuhrman grading [3,8,14]. Histogram-based features performed as a promising tool for differentiating RCC subtypes from RO [15]. However, none of the above studies mentioned the exclusion of ccRCC with necrosis and cystic lesions. Histological tumor necrosis is an independent prognostic indicator of ccRCC and pRCC [14].

The purpose of this study was to evaluate the feasibility of the whole-lesion CT-based texture analysis in identifying homogeneous renal tumors (including ccRCC, chRCC, and RO) without hemorrhage, necrotic, or cystic changes.

## 2. Materials and Methods

### 2.1. Patients

**Figure 1:**
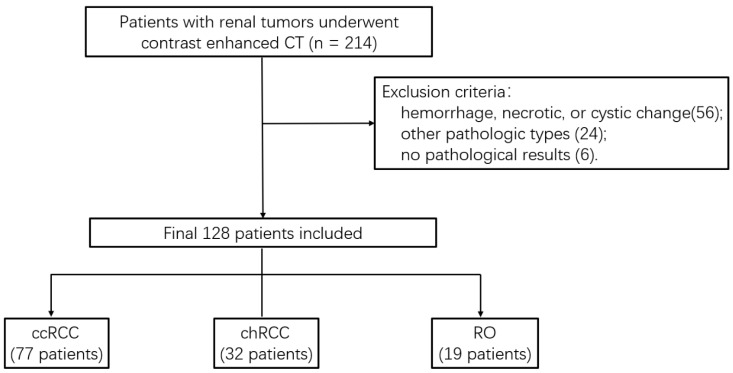
Flowchart of the study population.

This retrospective study was approved by our IRB, and written informed consent was waived. Imaging data from the reporting system of our hospital between July 2012 and August 2017 were retrospectively reviewed. According to the inclusion criteria, patients were identified as follows: (1) tumors confirmed by surgical histology (ccRCC, chRCC, and RO) without treatment before examination; (2) the interval between CT enhancement and operation is less than one month; (3) CT images showed without obvious artifacts or breathing motion artifacts; (4) tumors displayed no obvious hemorrhage, necrotic, or cystic changes. The exclusion criteria were as follows: (1) tumor showed hemorrhage, necrotic, or cystic changes (*n* = 56); (2) pathological results conformed other pathologic types (*n* = 24); (3) tumor had no pathological results (*n* = 6). Finally, 128 patients (77 ccRCCs, 32 chRCCs, and 19 ROs) were included in this study (Figure 1).

### 2.2. CT Examination

All patients underwent contrast-enhanced CT examination on 64-channel multi-detector CT scanners including HD 750 MDCT scanner (High Discovery 750, HD750, GE Medical System, Chicago, IL, USA) (*n* = 76) and Lightspeed VCT (GE Medical System, USA) (*n* = 52). All patients were in a supine position and feet first. The scanning protocol included arterial phase, venous phase, and delayed phase. The scan range covered the entire renal region area, and each phase of the scan was completed during a breath-hold. Nonionic contrast agents (iopromide, Ultravist 370; Schering, Berlin, Germany) were injected into median cubital vein through an 18-gauge (18-G) angiographic catheter at a rate of 3–4 mL/s using an automatic dual-flow high-pressure injector (Stellant; Medrad, Indianola, PA, USA). The total dose of contrast agent varied according to the patient’s body weight (1.5 mL/kg) and followed with 20 mL saline solution flush. The image was acquired by helical acquisition with parameters of 120 kV, automatic mA, helical pitch of 0.983, rotation speed of 0.6 s, slice thickness and interval of 5 mm/5 mm, and the thickness of reconstruction slice was 1.25 mm. The arterial phase was automatically trigged by monitoring the aortic density value (120 HU). After arterial phase scan, the venous phase was delayed by 25 s, and after 3 min, the delayed phase was performed.

### 2.3. Imaging Analysis

Patient information was removed from the image of the study, and histogram analysis was performed, which is a first-order texture analysis without filtering using histogram methods. All arterial and venous DICOM data were transmitted from the picture archiving and communication systems (PACS) to a personal computer, and texture analysis was performed with a developed software (Fire Voxel, New York University, New York, NY, USA). The radiologist (with 7 years of work experience in abdominal imaging diagnosis) manually delineated the area of interest (ROIs) of the whole layer of tumor as large as possible along the edge of the lesion, avoiding perirenal fat. Then, the ROI of each layer was fused to obtain the whole-lesion information of the lesion. Texture analysis parameters such as mean, inhomogeneity, skewness, kurtosis, and entropy were automatically generated, and then statistical software was used to obtain the 25th, 50th, and 75th percentile values.

### 2.4. Statistical Analysis

Statistical analysis was performed using SPPS 22.0 (Chicago, IL, USA) and MedCalc (MedCalc Software, Mariakerke, Belgium) statistical software for Windows. All the texture analysis parameters were recorded as mean ± standard deviation (std). Bonferroni corrections was used for one-way ANOVA to compare the parameters mean, 25th, 50th, 75th percentile, inhomogeneity, skewness, kurtosis, and entropy between ccRCC and chRCC, ccRCC and RO, and chRCC and RO both in the arterial phase and venous phase. The diagnostic performance of each parameter was calculated by receiver operating characteristic (ROC) curve analysis, and the differences between AUCs were estimated by DeLong test. A *p*-value lower than 0.05 indicated a statistically significant difference.

## 3. Results

**Figure 2:**
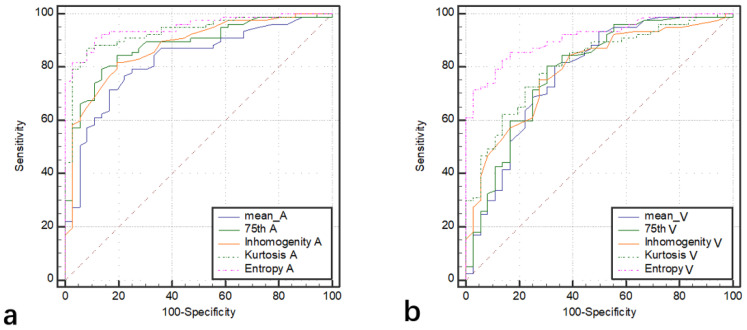
The ROCs of texture analysis parameters (mean, 75th, inhomogeneity, kurtosis, and entropy) in differentiating ccRCC from chRCC in arterial phase (**a**) and venous phase (**b**).

**Figure 3:**
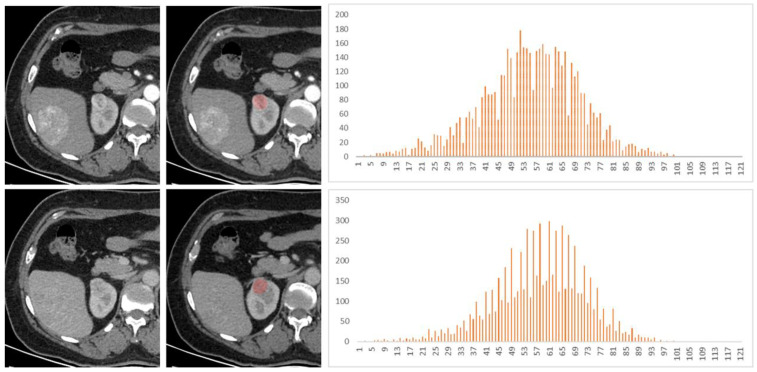
A 53-year-old female patient had a right renal tumor, which was pathologically confirmed as clear cell renal cell carcinoma (ccRCC). The liver tumor was pathologically confirmed as hepatic angiomyolipoma. The first and second lines of images were arterial phase and venous phase scans, respectively. Texture analysis parameters were 119 HU, 0.030, −0.39, −0.05, and 4.18 in arterial phase and 105 HU, 0.020, −0.61, 1.22, and 3.74 in venous phase (mean, inhomogeneity, skewness, kurtosis, and entropy).

**Figure 4:**
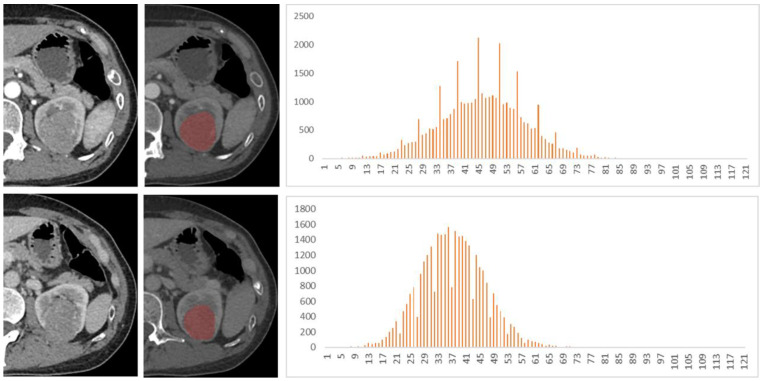
A 48-year-old female patient with left renal tumor was pathologically confirmed as chromophobe renal cell carcinoma (chRCC). The first and second lines of images were arterial phase and venous phase scans, respectively. Texture analysis parameters were 81 HU, 0.014, 0.29, −1.35, and 3.49 in arterial phase and 77 HU, 0.015, 0.10, 0.62, and 3.56 in venous phase (mean, inhomogeneity, skewness, kurtosis, and entropy).

**Figure 5:**
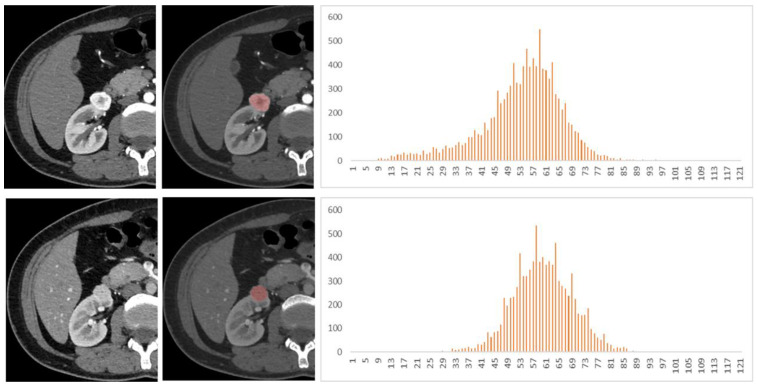
A 40-year-old female patient with a right renal tumor was pathologically confirmed as renal oncocytoma (RO). The first and second lines of images were arterial and venous phase scans, respectively. Texture analysis parameters were as follows: 272 HU, 0.042, −0.73, 0.89, and 3.94 in arterial phase and 154 HU, 0.018, −0.45, 2.91, and 3.53 in venous phase (mean, inhomogeneity, skewness, kurtosis, and entropy).

Among the 128 patients, there were 77 ccRCCs (M:F = 54:23, mean age 47.5 years), 32 chRCCs (M:F = 17:15, mean age 48.4 years), and 19 ROs (M:F = 12:7, mean age 53.5 years). The mean, 25th, 50th, 75th percentile, inhomogeneity, skewness, kurtosis, and entropy are shown in Table 1, and the *p* values and AUCs are shown in Table 2 and Table 3 and Figure 2, respectively. The histograms of representative cases of ccRCC, chRCC, and RO are shown in Figure 3, Figure 4 and Figure 5, respectively.

### 3.1. ccRCC vs. chRCC

The mean, 25th, 50th, 75th percentile, inhomogeneity, and entropy of ccRCC were significantly higher than those of chRCC, and the kurtosis was significantly lower than that of chRCC (all *p* < 0.05). The AUCs of 75th percentile, inhomogeneity, and kurtosis in arterial phase (0.88, 0.90, and 0.92) were significantly higher than those in the venous phase (0.79, 0.81, and 0.80) (all *p* < 0.05). The AUCs of mean, 25th, and 50th percentile in the arterial phase were higher than those in the venous phase, but the differences were not statistically significant (*p* > 0.05). The entropy yielded the highest diagnostic performance with AUC, sensitivity, and specificity of 0.95, 91%, and 89% in the arterial phase and 0.91, 83%, and 86% in the venous phase, respectively.

### 3.2. ccRCC vs. RO

The values of inhomogeneity of RO both in arterial and venous phases were significantly lower than those of ccRCC. The AUC (0.77) of inhomogeneity in the arterial phase was higher than that (0.66) in the venous phase, but the difference was not significant (*p* = 0.180). There were no significant differences in the remaining histogram parameters between the RO and ccRCC both in arterial and venous phases.

### 3.3. chRCC vs. RO

The entropy of RO was significantly higher than that of chRCC both in arterial and venous phases, and the AUC was 0.85 and 0.84, respectively. In the arterial phase, the kurtosis of RO (1.24 ± 1.50) was significantly lower than that of chRCC (4.97 ± 6.91) (*p* < 0.05), with an AUC of 0.84 (sensitivity 91%; specificity 74%), but there was no significant difference in the venous phase (*p* > 0.05). Other texture analysis parameters showed limited value in identifying chRCC from RO.

## 4. Discussion

In this study, we found that there were significant differences in texture analysis parameters including mean, 25th, 50th, 75th percentile, inhomogeneity, kurtosis, and entropy between ccRCC and chRCC. This result is in line with a previous study [16]. The inhomogeneity plays an important role in distinguishing RO from ccRCC. Entropy and kurtosis provide valuable information for distinguishing RO from chRCC.

Several previous studies have only described the value of texture analysis in distinguishing chRCC from other renal tumors, not in discriminating ccRCC from chRCC. Yu et al. [15] found that in the histogram parameters, only the parameter STD5 showed a discriminator with an AUC of 0.76 between chRCC and other renal tumors. Histogram analysis explained only about 64% of texture differences between chRCC and other renal tumors [16]. Although chRCC and ccRCC have substantial overlap in enhancement, chRCC tends to have lower and more homogenous enhancement than ccRCC [6]. Entropy reflects the complexity of an image, kurtosis reflects the peak value or sharpness of pixel distribution, and inhomogeneity reflects the inhomogeneity the pixel distribution [14]. In contrast to ccRCC, chRCC is most likely to be a homogeneous mass with a relatively hypovascular enhancement and tends to present attenuation peaks in the arterial or venous phase. On the contrary, ccRCC tends to show peak enhancement in the arterial phase and with washout in the venous phase. However, chRCC has a large overlap in appearance compared to ccRCC [17,18]. Therefore, our results can provide great help in discriminating homogeneous ccRCC from chRCC.

Challenges remain in distinguishing RO from ccRCC, especially homogeneous ccRCC. In our study, only the inhomogeneity had limited diagnostic performance in the arterial phase, while other parameters had limited help in both the arterial and venous phases, and these results were consistent with many reports in the literature. Central scarring and segmental enhancement inversion were considered unreliable and were controversial distinguishing features of RO from ccRCC [6,19]. Sasaguri, K and Takahashi [6] combined imaging features, such as tumor CT attenuation values and texture parameters (heterogeneity and skewness), to differentiate RO from RCC on biphasic contrast-enhanced CT. The results showed that this method could differentiate RO from ccRCC and other subtypes of RCC with an AUC of 0.82. Varghese et al. found that histogram analysis could only explain about 74% of the textural difference between RO and other tumors, and the AUC of the full texture model was 0.87 in distinguishing RO from ccRCC [16]. In addition, heterogeneity was an important parameter for renal tumor identification. RO was more homogenous than RCC [7]. Yu et al. indicated that the GLCM based on machine-based learning was a very good discriminator, with an AUC of 0.80, and kurtosis and skewness were excellent discriminators of ccRCC and RO, with AUCs of 0.93 and 0.91. In this study, the ROI drawn by the author included the entire tumor, and cystic necrosis of ccRCC could not be excluded [15].

It was difficult to differentiate RO from chRCC because of the same cell origin, even with a core needle biopsy. In our study, the entropy of arterial and venous phases and the kurtosis of the arterial phase demonstrated good diagnostic performance for discriminating chRCC and RO. This was inconsistent with the results of a previous study by Yu et al. They demonstrated that only the median showed a very good discriminator of chRCC from RO, with an AUC of 0.88, while the discriminators with other parameters were poor to fair [15]. Varghese et al. observed that the full texture model showed excellent discrimination in differentiating RO from chRCC, with an AUC of 0.94 [16]. However, the calculation of the above studies was relatively complex [14], while the histogram parameters based on ordinary CT images were simple and convenient, which could objectively quantify the heterogeneity of tumors.

There are several limitations in this study. First, this is a single-center retrospective study, and the results require further prospective validation with a large sample size. Second, the sample size of RO is relatively small, and the subtypes of the renal tumors included in this study are limited. Third, this study did not conduct interobserver consistency analysis, as previous studies have shown that the interobserver consistency of the whole tumor texture analysis is superior to the ROI of a single slice [20], and the interobserver consistency is excellent in several whole-tumor texture analysis studies [21].

In conclusion, the whole-lesion CT texture analysis, as a quantitative biomarker, could be a useful tool for characterizing a homogeneous renal tumor in contrast-enhanced CT images and in distinguishing ccRCC from chRCC and chRCC from RO, especially in the arterial phase. In addition, the results suggested that it is still challenging in distinguishing homogeneous ccRCC from RO with texture analysis.

## Figures and Tables

**Table 1:** Texture analysis parameters of the three groups of homogeneous renal tumors in arterial and venous phases.

		Mean	25th	50th	75th	Inhomogeneity	Skewness	Kurtosis	Entropy
Arterial phase	ccRCC	127 ± 50	91 ± 43	125 ± 50	159 ± 54	0.044 ± 0.011	−0.20 ± 0.48	0.70 ± 1.35	3.77 ± 0.21
	chRCC	83 ± 24	61 ± 22	80 ± 24	98 ± 26	0.028 ± 0.007	−0.71 ± 1.12	4.97 ± 6.91	3.25 ± 0.29
	RO	114 ± 61	84 ± 52	111 ± 61	135 ± 67	0.034 ± 0.010	−0.30 ± 0.48	1.24 ± 1.50	3.65 ± 0.29
Venous phase	ccRCC	140 ± 52	106 ± 44	138 ± 51	164 ± 56	0.038 ± 0.011	−0.47 ± 0.46	1.40 ± 1.89	3.72 ± 0.26
	chRCC	100 ± 46	77 ± 44	97 ± 47	115 ± 48	0.028 ± 0.007	−0.33 ± 0.70	3.65 ± 2.83	3.22 ± 0.35
	RO	130 ± 49	103 ± 46	128 ± 49	148 ± 51	0.031 ± 0.010	−0.70 ± 0.56	2.37 ± 2.53	3.64 ± 0.23

Note: the units of mean, 25th, 50th, 75th are HU (Hounsfield unit).

**Table 2:** The *p* values of texture analysis parameters in differentiating the three subtypes of homogeneous renal tumors in arterial and venous phases.

	Arterial Phase			Venous Phase		
	ccRCC vs. chRCC	ccRCC vs. RO	RO vs. chRCC	ccRCC vs. chRCC	ccRCC vs. RO	RO vs. chRCC
Mean	<0.001	0.767	0.080	0.001	1.000	0.116
25th	0.002	1.000	0.186	0.006	1.000	0.113
50th	<0.001	0.748	0.070	<0.001	1.000	0.103
75th	<0.001	0.202	0.056	<0.001	0.813	0.106
Inhomogeneity	<0.001	0.001	0.133	<0.001	0.013	1.000
Skewness	1.000	1.000	1.000	1.000	0.363	0.160
Kurtosis	<0.001	0.525	<0.001	<0.001	0.112	0.385
Entropy	<0.001	0.081	<0.001	<0.001	0.634	<0.001

Note: the units of mean, 25th, 50th, 75th are HU (Hounsfield unit).

**Table 3:** Discrimination power (AUC) of texture analysis parameters in differentiating the three subtypes of homogeneous renal tumors in arterial and venous phases.

		Arterial Phase		Venous Phase		
		AUC (Sensitivity, Specificity)	Cut-Off Value	AUC (Sensitivity, Specificity)	Cut-Off Value	*p*
ccRCC vs. chRCC	Mean	0.82 (71%, 83%)	100	0.78 (81%, 67%)	94	0.13
	25th	0.76 (65%, 78%)	70	0.74 (78%, 64%)	71	0.60
	50th	0.83 (78%, 78%)	93	0.78 (82%, 67%)	91	0.09
	75th	0.88 (79%, 86%) *	119	0.79 (81%, 70%) *	114	0.002
	Inhomogeneity	0.88 (82%, 81%) *	0.033	0.79 (75%, 72%) *	0.031	0.001
	Kurtosis	0.93 (87%, 92%) *	1.31	0.81 (73%, 78%) *	1.87	0.004
	Entropy	0.95 (91%, 89%)	3.54	0.91 (83%, 86%)	3.56	0.05
ccRCC vs. RO	Inhomogeneity	0.77 (61%, 90%)	0.039	0.66 (92%, 47%)	0.025	0.18
RO vs. chRCC	Kurtosis	0.84 (92%, 74%)	1.34	-	-	-
	Entropy	0.85 (92%, 74%)	3.56	0.84 (97%, 58%)	3.62	0.92

Note: the units of mean, 25th, 50th, 75th are HU (Hounsfield unit). * indicates a statistically significant difference between arterial AUC and venous AUC.

## Data Availability

Not applicable.
